# Oxytocin and social gaze during a dominance categorization task in tufted capuchin monkeys

**DOI:** 10.3389/fpsyg.2022.977771

**Published:** 2022-09-20

**Authors:** Meghan J. Sosnowski, Fumihiro Kano, Sarah F. Brosnan

**Affiliations:** ^1^Department of Psychology, Georgia State University, Atlanta, GA, United States; ^2^Language Research Center, Georgia State University, Atlanta, GA, United States; ^3^Centre for the Advanced Study of Collective Behaviour, University of Konstanz, Konstanz, Germany; ^4^Kumamoto Sanctuary, Kyoto University, Kumamoto, Japan; ^5^Max Planck Institute of Animal Behavior, Radolfzell, Germany; ^6^Center for Behavioral Neuroscience, Georgia State University, Atlanta, GA, United States; ^7^Neuroscience Institute, Georgia State University, Atlanta, GA, United States

**Keywords:** capuchin, non-human primate, eyetracking, oxytocin, social knowledge

## Abstract

Visual attention to facial features is an important way that group-living primate species gain knowledge about others. However, where this attention is focused on the face is influenced by contextual and social features, and emerging evidence in *Pan* species suggests that oxytocin, a hormone involved in forming and maintaining affiliative bonds among members of the same group, influences social attention as measured by eye gaze. Specifically, bonobos tend to focus on conspecifics’ eyes when viewing two-dimensional images, whereas chimpanzees focus more on the edges of the face. Moreover, exogenous oxytocin, which was hypothesized to increase eye contact in both species, instead enhanced this existing difference. We follow up on this to (1) determine the degree to which this *Pan* pattern generalizes across highly social, cooperative non-ape primates and (2) explore the impact of exogenously administered vs. endogenously released oxytocin in impacting this behavior. To do so, we tracked gaze direction on a computerized social categorization task using conspecific faces in tufted capuchin monkeys (*Sapajus [Cebus] apella*) after (1) exogenously administering intranasal oxytocin using a nebulizer or (2) inducing an endogenous increase in oxytocin using fur-rubbing, previously validated to increase oxytocin in capuchins. Overall, we did not find a general tendency in the capuchins to look toward the eyes or mouth, but we found that oxytocin was related to looking behavior toward these regions, albeit not in a straightforward way. Considering frequency of looking per trial, monkeys were more likely to look at the eye region in the fur-rubbing condition as compared to either the saline or exogenous oxytocin conditions. However, in terms of duration of looking during trials in which they did look at the eye region, monkeys spent significantly less time looking at the eyes in both oxytocin conditions as compared to the saline condition. These results suggest that oxytocin did not necessarily enhance eye looking in capuchins, which is consistent with the results from *Pan* species, and that endogenous and exogenous oxytocin may behave differently in their effect on how social attention is allocated.

## Introduction

As highly social species for whom vision is the dominant sensory modality, primates rely on visual cues to navigate their social world (Emery, 2000). The ability to quickly and accurately assess another individual visually is a critical part of deciding how to respond to that conspecific, and appropriate or inappropriate behavior will affect ongoing relationships that are important to maintaining social stability. Primates, as well as other species, may use gaze direction and physical orientation to make predictions about another’s intentions or to communicate their own intentions to a conspecific. The primate brain is especially well-equipped to do so – prior research has pinpointed brain regions specifically responsible for assessing gaze direction of a conspecific in both human (Carlin et al., 2011) and non-human primates (rhesus macaques, *Macaca mulatta*: Marciniak et al., 2014), an ability that provides information to the observer about the conspecific’s focus of attention. In addition, primates appear to tailor their allocation of social attention to the features of a conspecific that are most relevant to their social world, as demonstrated by species-level differences in which features of a conspecific, particularly facial features, elicit the most attention. Even when species are closely related, as in the *Pan* species, differing social structures elicit differences in attention to facial features – bonobos (*Pan paniscus*) attend to eye regions when viewing full-body videos and images of conspecifics more than do chimpanzees (*Pan troglodytes*), which view mouth, target-object-action regions, and genital areas to a greater degree than the eyes (Kano et al., 2015). This is hypothesized to be because bonobos are more motivated by social affiliation and coordination, a difference which might be related to greater attention to conspecifics’ eye regions than in their chimpanzee counterparts (Kano et al., 2015).

There are also contextual and individual factors that drive in-the-moment allocation of attention, especially those factors that are already linked to social affiliation. Oxytocin, a hormone that has been widely connected to affiliation, plays a role in visual social cognition by directing attention to socially relevant stimuli (for a review, see Shamay-Tsoory and Abu-Akel, 2016). For instance, exogenously administered oxytocin increased attention to the eye region of 2D images when presented to human subjects (Guastella et al., 2008), although this effect seems to be most prevalent when viewing familiar faces as opposed to unfamiliar ones (Marsh et al., 2021), and rhesus macaques showed a similar increase in the amount of time spent looking at the eyes as compared to a size-matched mouth region (Dal Monte et al., 2014), which would seem to suggest that oxytocin is enhancing eye contact in these species. However, intriguing evidence for species-level differences in this effect comes from a recent study comparing the two *Pan* species, in which researchers presented chimpanzees and bonobos with videos and still images of conspecifics following exogenous oxytocin administration. In this study, rather than enhancing attention to the eye-region across both species, oxytocin exacerbated the existing species tendencies; chimpanzees tended to attend even more to the mouth region of photos while bonobos showed significantly more eye contact than baseline (Kano et al., 2015; Brooks et al., 2021), suggesting that not only might species differ in overall tendencies toward eye contact, but also that oxytocin might influence this tendency differently in species with different social structures.

The relationship between attention to specific facial features and oxytocin has not been well-studied in non-ape primates other than in rhesus macaques. However, like the *Pan* species, other primates live in large, mixed-sex social groups in which they must manage complex social relationships, suggesting that visual social attention might be similarly important for them. One such species is the tufted capuchin monkey (*Sapajus apella*), a gregarious South American monkey that lives in large, mixed-sex social groups. Like chimpanzees and bonobos, tufted capuchin monkeys have been shown to change their behavior following both exogenous oxytocin administration and endogenous oxytocin release (Benítez et al., 2018; Sosnowski et al., in review; although see also Brosnan et al., 2015). Further, tufted capuchin monkeys not only recognize familiar conspecifics (Talbot et al., 2016), but also categorize familiar and unfamiliar conspecifics into dominance ranks based on structural facial features (Meachem et al., in revision, indicating that social gaze is an important part of their assessment of others. However, it is unknown which features of a conspecific’s face are important to capuchins when making these assessments, or if, as with chimpanzees and bonobos, this directed social attention is related to oxytocin levels. One hypothesis is that, given their male-dominated social structure, in which other male capuchins are a threat and therefore males tend to respond more strongly in intergroup encounters (Scarry, 2017), as is also true in chimpanzees (Wilson and Wrangham, 2003), capuchins might, like chimpanzees, attend equally to the eye and mouth region (Kano et al., 2015; Brooks et al., 2021). Alternatively, because both sexes of capuchin monkeys form ongoing affiliative bonds with both same- and opposite-sex conspecifics within their social group (Fragaszy et al., 2004b), they might, like bonobos, attend significantly more to the eye region (Kano et al., 2015; Brooks et al., 2021).

Finally, all of the prior research on the relationship between oxytocin and social attention has used exogenously administered oxytocin. But of course, in normal interactions, oxytocin is released endogenously, and it has been suggested that exogenous oxytocin may influence behavior differently than endogenously produced oxytocin (Insel, 1992), or that administering a concentration of oxytocin above biologically relevant levels might change the relationship between oxytocin and behavioral outcomes (Crockford et al., 2014). Despite these potential differences, no literature has directly manipulated both exogenous and endogenous oxytocin to compare how they affect social behavior or attention, and very few even measure non-manipulated endogenous levels to compare their effects with those of exogenous oxytocin (for a review, see Bartz et al., 2011). This is likely because it is often difficult to reliably induce oxytocin increase *via* natural social behavior, especially without confounding effects of social dynamics within a group (i.e., using grooming to induce oxytocin release).

We had a unique opportunity to do so in tufted capuchin monkeys, which show increased urinary oxytocin levels following an easily-induced, species-typical behavior, fur-rubbing (Benítez et al., 2018; Sosnowski et al., in review). Tufted capuchin monkeys reliably engage in a fur-rubbing, or anointing, behavior when presented with pungent materials, such as onions, some plant materials, or insects (Alfaro et al., 2012; Bowler et al., 2015). Individuals often congregate with conspecifics in their social group to do so in concert and in contact with others, suggesting that the behavior is part of their affiliative repertoire. However, capuchin monkeys will also reliably fur-rub when presented with these materials alone, and importantly, this solo-fur-rubbing increases oxytocin similarly to fur-rubbing in concert with groupmates (Sosnowski et al., in review). Therefore, fur-rubbing (either socially or individually) is a validated method for experimentally increasing endogenous oxytocin this species, which provided us the ability to experimentally manipulate both endogenous oxytocin and administer exogenous oxytocin for a direct comparison. By studying social gaze after both administering exogenous oxytocin and inducing the release of endogenous oxytocin, we hoped to assess if the two manipulations had similar behavioral effects.

To explore the relationship between social gaze and oxytocin in this species, we exposed tufted capuchin monkeys to one of three oxytocin manipulations: intranasally administered exogenous oxytocin, endogenously induced oxytocin (through capuchins’ natural behavior of fur rubbing), and a control condition in which capuchins received intranasal saline rather than an oxytocin manipulation. Then, we presented the monkeys with a previously-trained computerized task in which they needed to categorize male conspecific faces based on perceived dominance (Meachem et al., in revision); in the present study, our goal was not to determine how they categorized faces, but to see which features of the face monkeys chose to observe while completing the task. To do so, we recorded their gaze behavior to the eyes, the mouth, and the whole face using an unrestrained eye-tracking setup. Given the species-level behavior observed in the *Pan* species, we hypothesized that the oxytocin manipulations would exacerbate existing species-level preferences in the capuchin monkeys, although we did not make a prediction about the specific direction of this species-level preference with respect to looking at the eyes or mouth more overall.

## Materials and methods

### Subjects and study site

Our subjects were five tufted capuchin monkeys (three females, two males; age range: 13–23 years old) living in social groups at the Language Research Center (LRC) of Georgia State University (GSU) who were both trained for our unrestrained exogenous oxytocin nebulization and from whom we could get an unrestrained calibration on the eye-tracker. At the LRC, capuchins live in one of five multi-male, multi-female groups ranging in size between 4 and 9 individuals except one bachelor pair of males that lives adjacent to one of the other social groups (both their inside and outside enclosures are within 1 m of the other group, so aside from each other, they have visual and auditory access to conspecifics at all times). Each group is housed in their own indoor/outdoor enclosure; monkeys come inside in the morning to participate in cognitive testing, if they choose to do so, until early afternoon, at which time they are given access to their outdoor play yard for the remainder of the day, unless there is inclement weather. All monkeys are fed a species-appropriate diet of fruits, vegetables, and chow at several times throughout the day and have *ad libitum* access to water, including during cognitive testing; no monkey is ever deprived of food, water, or outdoor or social access for the purposes of encouraging participation in testing. All of the research reported in the manuscript was conducted in compliance with the procedures approved by the Institutional Animal Care and Use Committee (#A16031, #A18047) of Georgia State University, as well as all laws and practices governing the study of non-human primates in the United States of America.

Monkeys are trained to voluntarily enter an individual testing chamber to participate in cognitive and behavioral testing; no monkey is ever restrained and all testing is non-invasive. There are no consequences for choosing not to separate, other than an inability to participate in the cognitive testing for the day. Once they are separated, monkeys routinely participate in both manual and computerized testing using the LRC Computerized Testing system, which consists of a computer (either desktop or laptop), a monitor, an automated pellet dispenser, and modified joystick. All of the subject monkeys are familiar with the computerized testing setup, with at least 3 years of experience working on computer programs for cognitive testing.

### Apparatus and calibration

**FIGURE 1:**
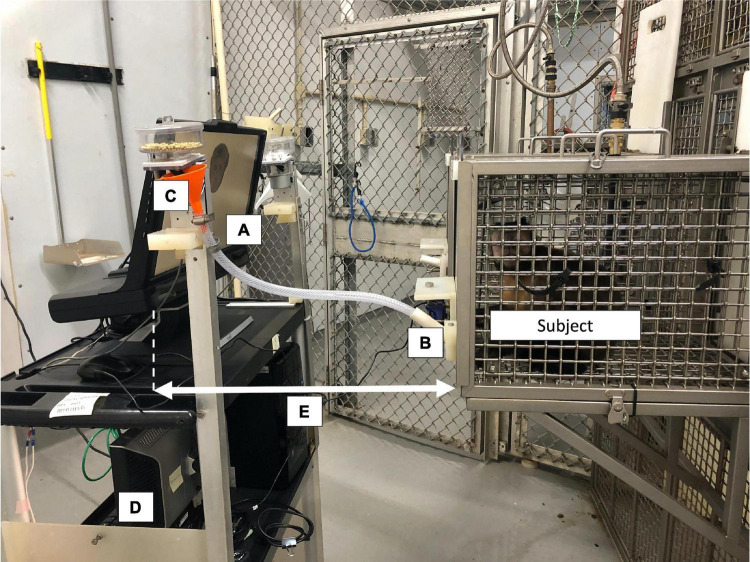
Example of the eyetracker setup used in the present study. The cognitive task was displayed on a monitor **(A)** attached to the Tobii TX300 eyetracker. The subject interacted with the task using a modified joystick **(B)** and was rewarded automatically for correct answers using a pellet dispenser **(C)**. Researchers used a second monitor facing the opposite direction **(D)** during calibration to advance the procedure point-by-point. The distance between the monkey’s faceplate and the eyetracker **(E)** remained consistent within an individual throughout calibrations and all testing sessions. This photo was taken with the overhead room lights on for better visibility; however, all calibrations and testing sessions occurred with the overhead lights off.

The experimental task (see below) was programmed and run in Python 2.7 on the Windows Vista operating system. The present study used the monkeys’ typical computerized testing system, to which we connected an infrared head-free eye tracker (300 Hz, TX300, Tobii Technology) with associated monitor (resolution 1920 × 1080). We also used Tobii Studio software (Tobii Technology) to set up, calibrate, and record the screen during the experimental task on the desktop computer connected to the eyetracker (Figure 1). We conducted all eyetracking procedures (calibration and testing) in a semi-dark room (the overhead fluorescent lights were turned off prior to eyetracking onset, but we were unable to control for the fact that windows in each room allowed in a small amount of natural light).

For each subject monkey prior to testing, we achieved an acceptable two-point calibration using the infant calibration settings included in Tobii Studio, as used in previous studies with unrestrained primates (Kano and Tomonaga, 2009, 2011; Brooks et al., 2021). To orient the monkey’s attention prior to the calibration, we used a still image of a start button, which appears in many of their computerized cognitive tasks to indicate the start of a trial. When the researcher (MS) saw that the monkey was attending to the screen, the researcher manually pressed the space bar to move into the first point of calibration, in which a small animation and sound provided by the Tobii Studio software was presented at the top left corner on the screen. The researcher watched the monkey’s eyes for attention to the relevant portion of the screen. When the monkey appeared to be attending to the correct portion of the screen (usually within 1–2 s for a successful calibration), the researcher would again press the space bar to move to the second point of calibration, the bottom right corner. Again, the researcher would watch the monkey’s eyes for the change in their gaze, and when it appeared that the monkey was looking at the second calibration point, would press the space bar a final time to end the calibration. At this point, Tobii Studio would alert the researcher of the calibration quality, or alternatively, that there was not enough data for a successful calibration (in which case we would repeat the calibration procedure as needed). If the calibration was successful, we accepted the calibration and checked it using a series of icons on the screen to ensure that the monkey’s recorded gaze did not deviate significantly from the icons. No work has previously been done in eye tracking unrestrained capuchins in which the eyetracker was calibrated using the subjects themselves (although previous work in this species has used calibrations from human infants as a proxy: Howard et al., 2018; Lonsdorf et al., 2019), but similar live calibration procedures have been used in other primate species, resulting in reliable results in other species of monkey (although they used a 5-point calibration procedure: Paukner et al., 2014; Ryan et al., 2019) and in ape species (which used a 2-point calibration procedure similar to ours: Kano and Tomonaga, 2009; Kano et al., 2012) to within one-degree of reliability. Therefore, a successful calibration using our procedure should provide the accuracy needed to distinguish if the monkeys were looking at our regions of interest within the face stimuli. Each subject was calibrated once prior to beginning testing and this calibration was used for all of their testing sessions. We recorded the eyetracker angle and exact distance from the front of the testing chamber after each monkey’s successful calibration, as these differed per monkey due to the location of their testing chamber and the sloped floor (Angle: *M* = 5.4°, *SD* = 3.72°, *Min* = −1°, *Max* = 10°; Distance: *M* = 21.1′′, *SD* = 1.53′′, *Min* = 19′′, *Max* = 22.5′′). However, in order to ensure gaze precision across the multiple sessions within each subject, we used these individual specifications for all further testing sessions for each monkey.

### Oxytocin manipulation

On testing days, once monkeys were separated into their individual testing chambers, but prior to beginning the computer task on the eyetracker, each subject underwent one of three oxytocin manipulations: one in which the subject received exogenous oxytocin intranasally *via* a nebulizer, one in which we induced fur-rubbing in the subject by giving them access to onions, and a control condition in which subjects received saline intranasally *via* a nebulizer.

In the exogenous oxytocin condition, we administered oxytocin to each subject using a method that we previously developed for capuchin monkeys (Brosnan et al., 2015; Benítez et al., 2018). We trained the monkeys to target by holding onto a Kong toy attached to their enclosure with both hands; we then administered ∼15 IU crystallized New World Monkey oxytocin (Pro^8^ oxytocin, Anaspec; reconstituted in saline) to the subject using a NebPak handheld baby nebulizer. We administered the oxytocin in six ten-second bouts for a total of 60 s of administration, at a rate of 1 mL per minute; monkeys were rewarded with a one-inch cube of apple at the end of each successful bout. All subjects completed all exogenous oxytocin administration within 3 mins of the onset of the first bout. During administration, we visually monitored the nebulization process to ensure that monkeys were breathing in the nebulized oxytocin, as evidenced by a distinctive “puffing” of the visible vapor. After exogenous administration was complete, monkeys underwent a 15 min incubation period prior to the computer task or eyetracking, which was when we first saw a significant increase in urinary oxytocin in our prior work validating these methods (Benítez et al., 2018).

In the fur-rubbing condition, we induced an increase in endogenous (naturally-occurring) oxytocin by having the monkeys engage in a species-specific social behavior, fur-rubbing. This population of monkeys has previously been shown to reliably engage in social fur-rubbing behavior when given access to onions. Our previous work demonstrated that fur-rubbing increased urinary oxytocin comparably to exogenous administration (Benítez et al., 2018), but because the oxytocin is naturally produced in the brain itself, we were able to avoid the question of if the oxytocin crosses the blood-brain barrier to affect behavior, as is a concern with exogenous oxytocin, or correct dosage. Further, we also have demonstrated that there is no statistical difference in urinary oxytocin levels when capuchins fur-rubbed as a group versus when they fur-rubbed alone (Sosnowski et al., in review). Therefore, providing onions to the monkeys when already separated was an appropriate manipulation to increase endogenous levels of oxytocin that also allowed us to minimize disruptions and to control timing between fur-rubbing bouts and eyetracking. During this manipulation, we provided access to 1/4 onion per monkey while the monkeys were in their individual testing chambers. Each subject was allowed to fur-rub with the onions for up to 10 mins or until they lost interest (whichever came first). During the fur-rubbing bouts, we recorded start and end of fur-rubbing in order to ensure that monkeys did fur-rub during the 10-min allotted time (if they had not, we would not have used the session, but this did not occur). As in the exogenous oxytocin condition, we waited 15 mins after the end of fur-rubbing prior to beginning the computer task or eyetracking, as our previous work indicated that, again as with exogenous induction, oxytocin increased over the first 15 mins and remained elevated through at least the first 60 mins following fur rubbing (Benítez et al., 2018).

Finally, in our control condition, we followed the same procedure as in the exogenous oxytocin condition, but instead of nebulizing crystallized oxytocin, we nebulized saline. This allowed us to control for any effect of interacting with an experimenter in a manipulation that did not have increased oxytocin significantly in our previous work (Benítez et al., 2018). As in the two oxytocin manipulations, we waited 15 mins after the end of saline administration prior to beginning the task.

Monkeys completed two sessions in each of the above conditions, for a total of six sessions. Monkeys completed only one session in one condition per day.

### Task

Fifteen minutes after the day’s oxytocin manipulation, the experimenter started the testing program and immediately left the room (to remove the possibility of experimenter cuing) while monkeys completed a dichotomous choice categorization task that they were familiar with as they had recently experienced it for another study (Meachem et al., in revision; note that we collected new data for the current study, however as we used the same stimuli as in the previous task, the images themselves were no longer novel). In the task, they categorized images of male conspecific faces into “dominant” and “subordinate” categories, although for our purposes the goal was simply to give them a task for which they focused on conspecific faces; that is, we were not interested in their choices *per se*, just where the monkeys looked.

First, monkeys moved their cursor to a start square, after which a sample face stimulus appeared alone on the screen for 2 s. Then, the stimulus remained on the screen while monkeys made a determination of dominance ranking by using their joystick to move the computer cursor onto one of two symbols. One symbol, a blue triangle, had been previously trained to be the “dominant” category, while the other (a yellow cross) represented the “subordinate” category using images of familiar individuals from their social group. Prior to testing, monkeys were required to achieve a criterion of at least 80% correct categorizations on known conspecific faces; unknown conspecific faces were rewarded no matter which category the monkeys chose.

Images were presented on the eyetracker monitor’s screen, which had a resolution of 1920 × 1080. These images featured both familiar individuals (that is, males living at the LRC that the subjects saw regularly) and unfamiliar individuals (males from other research facilities, whom our subjects had never encountered before). All images consisted of a single male conspecific face from a head-on, frontal view with a neutral expression. Each image had been cropped to include only the face, ears, and “tuft” region (areas of longer hair at the top of the head) of the individual. We used 90 images (45 of familiar individuals, 45 of unfamiliar individuals) from 18 conspecific male individuals (9 familiar, 9 unfamiliar) in total, each at a size of 600 × 600 pixels. Images were randomly ordered and presented throughout the session, so monkeys observed different images at different times throughout the session. Again, because this was a familiar task, these images were no longer novel to the monkeys.

The monkeys were allowed to work on this task for 30 mins, during which time they were allowed to complete as many trials as they could within the testing session. During this time, we recorded their gaze data using the eyetracker using the “Screen Record” function included in Tobii Studio. A short video depicting two computerized trials as well as the associated eyetracker gaze recording is available in Supplementary material.

### Data handling

We chose to include up to the first 20 trials of each session to ensure that monkeys remained motivated to earn reward pellets, to avoid the possibility of satiation effects with respect to the amount of food being eaten, and to ensure that all sessions had comparable trial numbers (i.e., if not all subjects completed all trials in a session). This typically reflected about 3–4 mins of testing time in the session (*M*_*Seconds*_ = 318.44 or 5 mins and 18 s, *Median*_*Seconds*_ = 234.99 or 3 mins and 55 s, *SD*_*Seconds*_ = 248.22, *Min_*Seconds*_* = 183.06, *Max*_*Seconds*_ = 1228.54), meaning that these trials captured behavior from 25 to 28 mins after the onset of the oxytocin manipulation (that is, 15–18 mins after the end of the manipulation period); given that our previous work showed that urinary oxytocin significantly increased in this species beginning 15–30 mins after exogenous oxytocin administration (Benítez et al., 2018), we expected this time frame to capture any social gaze differences as the result of the oxytocin manipulation. Importantly, this roughly 3–4 min time period was also analogous to the time period of data collection in the previous study from *Pan* species, in which subjects viewed stimuli for 3–6 mins in total (Brooks et al., 2021). We also wanted to ensure that monkeys were actually motivated to work on the task, so if a monkey completed fewer than 10 trials within the 30 min session, we did not include that session in our dataset (see “Results” section).

**FIGURE 2:**
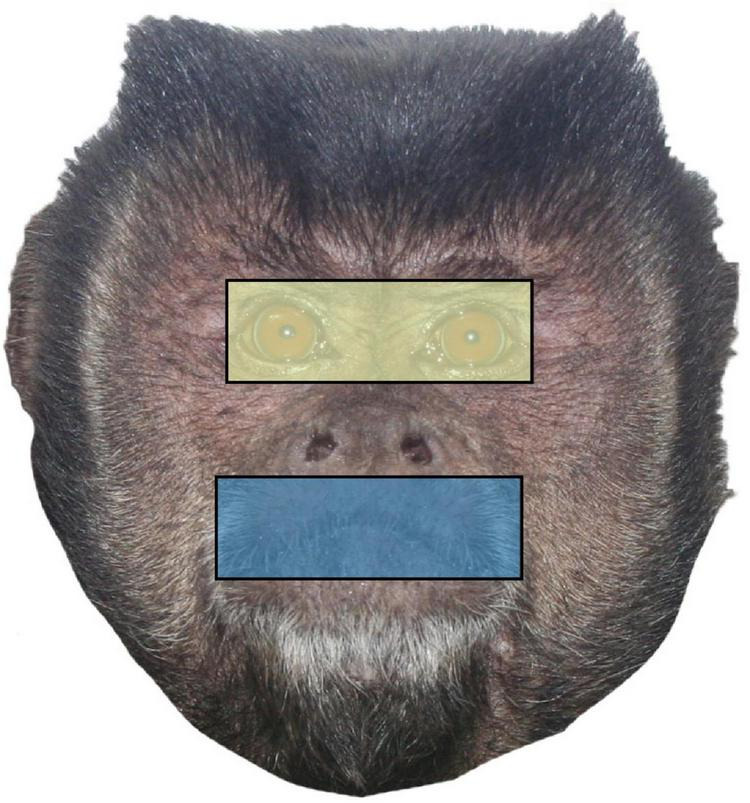
Sample face stimulus with the two conspecific regions (eyes and mouth) and the whole face identified as areas of interest (AOIs). Eye and mouth regions are size matched by pixel width and height.

For each trial, we manually identified areas of interest (AOIs) using the Tobii Studio software during the 2 s for which each face stimulus was alone on the screen (Figure 2). For each image, we first identified a region encompassing the eyes from corner to corner and top to bottom, which resulted in a rectangle. Then, we created a size matched mouth region that we centered over the mouth. Finally, because we wanted to assess each of these regions as a portion of the time spent looking at the whole face, we also included an all-encompassing rectangular region that captured the entire face. We then used Tobii Studio to calculate the amount of time in milliseconds spent looking at the whole face, the eye region, and the mouth region of each trial individually.

### Statistical analysis

Because there might be some effect of the specific stimuli that were randomly presented in each session, or of the familiarity of the conspecific pictured in those stimuli, we analyzed our data at a trial level. We began by assessing the trial-level dataset for assumptions of linear models using QQ-plots and histograms and by inspecting the dataset visually.

First, we wanted to compare the amount of time spent looking at the eye region on each trial to the amount of time spent looking at the mouth region. Because our looking time measures were generally extremely positively skewed, we used a Wilcoxon signed-rank test to first compare the amount of time spent looking at the eyes to the amount of time spent looking at the mouth. Then, we were interested in assessing how this relationship might change based on oxytocin condition. To do so, we calculated a difference score for each trial by subtracting the looking time to the mouth (in milliseconds) from the looking time to the eyes. Therefore, a positive difference score indicated that the monkey looked more at the eyes than at the mouth on that trial, while a negative difference score indicated the opposite. Because we were only interested in trials where monkeys looked at one or both of the regions, we removed trials in which they looked at neither the eyes nor the mouth. We then inspected this difference score measure using QQ-plots and histograms, and found it to be normally distributed and that it met the assumptions for linear modeling. Therefore, we built a linear mixed-model (LMM) using this difference score as an outcome measure, in which we included oxytocin condition as a fixed categorical predictor variable. In order to account for our repeated measures design, we included the random intercept term of subject, along with a second random intercept term of the lower-level factor of stimulus image nested within the higher-level factor of familiarity.

We then wanted to examine the looking behavior to each region individually to see if oxytocin condition impacted gaze behavior within those regions. In our dataset, the individual looking time measures for each AOI were severely positively skewed and zero-inflated, which made linear modeling inappropriate for these data. Therefore, we elected to use a hurdle model approach for each of our AOI outcome measures, which consisted of a two-part analysis for each outcome of interest. Hurdle models, although typically used for zero-inflated count data, have also been used for continuous outcomes like our ratio-level looking time durations (Baldwin et al., 2016; Boulton and Williford, 2018). Hurdle model frameworks typically consist of two questions: first, if there is an effect of the predictors on whether the outcome variable is zero or not, and second, when the outcome is not zero, if there is an effect of the predictors on the magnitude of the outcome. In other words, for each of our outcomes, we asked first if the monkeys looked at the region during the trial, and second, when their attention was captured by that region during a trial, for how long they looked at the region.

Using this framework, we wanted to analyze each AOI (whole face, eyes, and mouth) separately in order to see if there might be some effect of oxytocin condition on one region over another. To assess the likelihood of monkeys looking at the region, we coded a dummy binary outcome variable for each region that consisted of “looked” (1) or “did not look” (0). We then used this dummy variable as the outcome in a binomial mixed model for each region that included the fixed categorical predictor of oxytocin condition, for which the referent category was the saline condition. In each of these models, to account for our repeated measures design, we included subject as a random intercept. To the same end, we again included a second random intercept of stimulus image nested within familiarity. Therefore, for each region, our first model structure was: Looked ∼ Condition + (1| Subject) + (1| Familiarity/StimulusImage).

Then, as the second part of our hurdle model for each region, we analyzed how much time monkeys spent looking at the region during a trial based on condition. To do so, we subset the data to include only non-zero values for looking duration. We visually inspected these new datasets for assumptions of linear models using Q–Q Plots and histograms, and found them acceptable for linear modeling. Then, we fit a separate, second linear mixed-model using only these non-zero observations (those in which monkeys did look at the region) in which we predicted the effect of each of condition on the looking time to the region. As in our binary models, we included the random effects of subject and stimulus image nested within familiarity. Thus, our model structure for this part of our analysis for each region was Looking Time in MS ∼ Condition + (1| Subject) + (1| Familiarity/StimulusImage). We performed three hurdle model analyses using this framework: one for the whole face, one for the eye region, and one for the mouth region.

We compared each model to a null model consisting of the intercept and any random effect terms using a likelihood test. For each model, we conducted *post hoc* tests using estimated marginal means with a Satterthwaite correction for multiple comparisons to compare the likelihood of looking at the region in question in the endogenous oxytocin condition to the likelihood in the exogenous oxytocin condition. All analyses were run in R programming language in RStudio (R Development Core Team, 2016); the Wilcoxon signed-rank test was run using the “wilcox.test” function of the *stats* package. Mixed-models were built using the “glmer” and “lmer” functions of the *lme4* package (Bates et al., 2015), and likelihood tests were conducted using the “anova” function of the *stats* package.

## Results

In total, we collected 541 trials over 28 sessions from five subjects in total. Generally, monkeys did not spend significantly more time looking at the eye region over the mouth during each trial (in milliseconds: *M*_*Eyes*_ = 29.34, *SD_*Eyes*_* = 62.22, *Range*_*Eyes*_ = 0–437.80; *M*_*Mouth*_ = 30.24, *SD*_*Mouth*_ = 64.07, *Range*_*Mouth*_ = 0–492.10; *V* = 14,781, *p* = 0.714). In addition, there was no effect of oxytocin condition [*X^2^*(2) = 0.55, *p* = 0.758] on our calculated difference score measure (Table 1).

**TABLE 1:** Linear mixed-model predicting difference score from oxytocin manipulation.

	Difference score (eye–mouth)			
	
*Predictors*	*Estimates*	*SE*	*Conf. int. (95%)*	*p*
(Intercept)	−17.31	21.86	−60.16 to 25.54	0.428
Fur-rubbing	13.18	19.08	−24.22 to 50.57	0.490
Exogenous oxytocin	11.96	19.46	−26.19 to 50.10	0.539
N_Subject_	5			
Marginal *R*^2^/Conditional *R*^2^	0.002/0.002			

For the categorical predictor of oxytocin condition, the referent category is the saline condition. Significant estimates and their p-values are bolded. Full vs. null-model χ^2^(2) = 0.55, p = 0.760.

**FIGURE 3:**
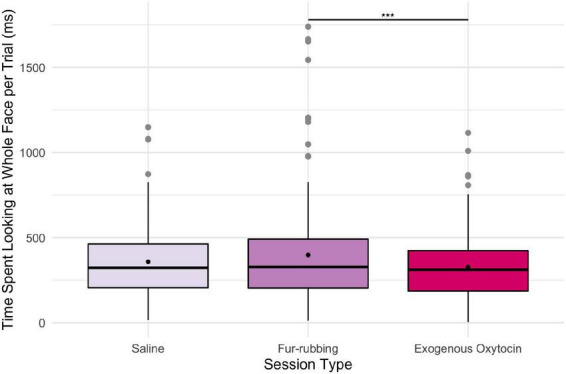
Boxplot of time (ms) spent looking at the whole face across the three oxytocin conditions on trials where monkeys were recorded looking at the regions. Black points represent overall means. ***p < 0.001.

In terms of looking behavior toward our specific AOIs, however, the oxytocin condition was a statistically significant predictor of the likelihood of looking at the stimulus face generally [*X^2^*(2) = 6.12, *p* = 0.047; Table 2A]. Monkeys were statistically significantly more likely to look at the face in the fur-rubbing condition (*Odds Ratio* = 1.96, *SE* = 0.59, CI = [1.09–3.53], *z* = 2.52, *p* = 0.02) than in the saline condition. Monkeys were not, however, either more or less likely to look at the face in the exogenous oxytocin condition (*Odds Ratio* = 1.03, *SE* = 0.28, CI = [0.60–1.76], *z* = 0.09, *p* = 0.925) than in the saline condition. There was also no difference in the likelihood of looking at the face between the fur-rubbing condition and the exogenous oxytocin condition (*β_*contrast*_* = 0.65, *SE* = 0.30, *z* = 2.14, *p* = 0.082). In the second part of our hurdle model analysis, when monkeys did look at the face, monkeys looked significantly longer at the whole face in the fur-rubbing condition as compared to the exogenous oxytocin condition (*β_*contrast*_* = 100.3, *SE* = 26.7, *t* = 3.76, *p* < 0.001). However, monkeys did not look at the whole face for significantly more or less time in the fur-rubbing condition as compared to the saline condition (β = 51.04, *SE* = 27.24, CI = [−2.34 to 104.42], *t* = 1.87, *p* = 0.061; Table 2B) or exogenous oxytocin condition as compared to the saline condition (β = −49.31, *SE* = 26.90, CI = [−102.03 to 3.41], *t* = −1.83, *p* = 0.067; Figure 3).

**TABLE 2:** Trial Level: Hurdle model analysis for gaze behavior at the whole face.

(A) Binary regression predicting likelihood of looking at the whole face				

	Likelihood of looking at region (whole face)			
	
*Predictors*	*Odds ratio*	*SE*	*Conf. int.* *(95%)*	*p*
(Intercept)	**7.16**	6.78	1.12 .145.78	**0.037**
Fur-rubbing	**1.96**	0.59	1.09 .03.53	**0.024**
Exogenous oxytocin	1.03	0.28	0.60 .61.76	0.925
N_Subject_	5			
Marginal R^2^/Conditional R^2^	0.013/0.545			

**(B) Linear mixed-model (LMM) of looking time duration to the whole face based on oxytocin condition**				

	**Looking duration (ms)**			
	
***Predictors***	***Estimates***	***SE***	***Conf. int. (95%)***	***p***

(Intercept)	**367.94**	71.57	227.68–508.21	**<0.001**
Fur-rubbing	51.04	27.24	−2.34 to 104.42	0.061
Exogenous oxytocin	−49.31	26.90	−102.03 to 3.41	0.067
N_Subject_	5			
Marginal R^2^/Conditional R^2^	0.025/0.323			

Full vs. null-model χ^2^(2) = 6.38, p = 0.041.

For the categorical predictor of oxytocin condition, the referent category is the saline condition. Significant estimates and their p-values are bolded. Full vs. null-model χ^2^(2) = 13.86, p < 0.001.

**FIGURE 4:**
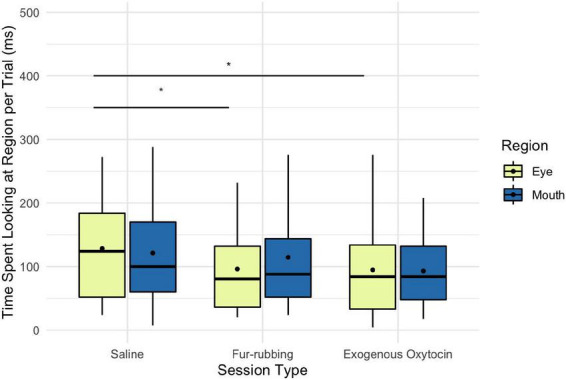
Boxplot of time (ms) spent looking at the eye and mouth regions across the three oxytocin manipulations on trials where monkeys did look at the regions. Black points represent overall means. *p < 0.05.

Monkeys were also more likely to look at the eye region of the stimulus image in the fur-rubbing (endogenous oxytocin) condition than in the saline condition (*Odds Ratio* = 2.13, *SE* = 0.57, CI = [1.26–3.60], *z* = 2.83, *p* = 0.005; Table 3A); the *post hoc* comparison between the fur-rubbing condition and the exogenous oxytocin condition was also significant (*β_*contrast*_* = 0.65, *SE* = 0.26, *z* = 2.48, *p* = 0.035), such that monkeys were more likely to look at the eye region in the fur-rubbing condition than the exogenous oxytocin condition. Monkeys were not significantly more or less likely to look at the eye region in the exogenous oxytocin condition as compared to the saline condition (*Odds Ratio* = 1.12, *SE* = 0.30, CI = [0.66–1.88], *z* = 0.42, *p* = 0.786). However, when monkeys did look at the eye region, we found that monkeys spent statistically significantly less time doing so in both the fur-rubbing condition (β = −31.28, *SE* = 15.01, CI = [−60.70 to −1.86], *t* = −2.08, *p* = 0.037; Table 3B) and the exogenous oxytocin condition (β = −34.69, *SE* = 15.93, CI = [−65.91 to −3.47], *t* = −2.18, *p* = 0.029) as compared to the saline condition (Figure 4); there was no significant difference between the amount of time spent looking at the eyes in the fur-rubbing condition as compared to the exogenous oxytocin condition (*β_*contrast*_* = 3.41, *SE* = 14.4, *t* = 0.24, *p* = 0.969).

**TABLE 3:** Trial level: Hurdle model analysis for gaze behavior at the eye region.

(A) Binary regression predicting likelihood of looking at the eye region				

	Likelihood of looking at region (eye region)			
	
*Predictors*	*Odds ratio*	*SE*	*Conf. int. (95%)*	*p*
(Intercept)	**0.22**	0.13	0.07–0.72	**0.012**
Fur-rubbing	**2.13**	0.57	1.26–3.60	**0.005**
Exogenous oxytocin	1.12	0.30	0.66–1.88	0.678
N_Subject_	5			
Marginal R^2^/Conditional R^2^	0.021/0.343			

**(B) Linear mixed-model (LMM) of looking time duration to the eye region based on oxytocin condition**				

	**Looking duration (ms)**			
	
***Predictors***	***Estimates***	***SE***	***Conf. int. (95%)***	***p***

(Intercept)	**125.81**	14.10	98.17.153.46	**<0.001**
Fur-rubbing	−**31.28**	15.01	−60.70 to −1.86	**0.037**
Exogenous oxytocin	−**34.69**	15.93	−65.91 to −3.47	**0.029**
N_Subject_	5			
Marginal R^2^/Conditional R^2^	0.038/0.038			

Full vs. null-model χ^2^(2) = 9.38, P = 0.009.

For the categorical predictor of oxytocin condition, the referent category is the saline condition. Significant estimates and their p-values are bolded. Full vs. null-model χ^2^(2) = 5.69, P = 0.058.

Finally, monkeys were no more or less likely to look at the mouth region as a result of the fur-rubbing condition (*Odds Ratio* = 1.30, *SE* = 0.33, CI = [0.79–2.13], *z* = 1.02, *p* = 0.309) or the exogenous oxytocin condition (*Odds Ratio* = 0.95, *SE* = 0.24, CI = [0.58–1.56], *z* = −0.18, *p* = 0.852) than they were in the saline condition (Table 4A); there was also no significant difference in the likelihood of looking at the mouth in the fur-rubbing condition as compared to the exogenous oxytocin condition (*β_*contrast*_* = 0.31, *SE* = 0.25, *z* = 1.22, *p* = 0.442). When monkeys did look at the mouth region, neither the fur-rubbing condition (β = −9.31, *SE* = 15.72, CI = [−40.13 to 21.51], *t* = −0.59, *p* = 0.554) nor the exogenous oxytocin condition (β = −26.90, *SE* = 15.65, CI = [−57.57 to 3.77], *t* = −1.72, *p* = 0.086) was significantly related to the amount of time spent looking at the mouth (Table 4B).

**TABLE 4:** Trial level: Hurdle model analysis for gaze behavior at the mouth region.

(A) Binary regression predicting likelihood of looking at the mouth region				

	Likelihood of looking at region (mouth region)			
	
*Predictors*	*Odds ratio*	*SE*	*Conf. int. (95%)*	*p*
(Intercept)	**0.31**	0.18	0.10–0.97	**0.044**
Fur-rubbing	1.30	0.33	0.79–2.13	0.309
Exogenous oxytocin	0.95	0.24	0.58–1.56	0.852
N_Subject_	5			
Marginal R^2^/Conditional R^2^	0.005/0.005			

**(B) Linear mixed-model (LMM) of looking time duration to the mouth region based on oxytocin condition**				

	**Looking duration (ms)**			
	
***Predictors***	***Estimates***	***SE***	***Conf. int. (95%)***	***p***

(Intercept)	**120.46**	13.20	94.58–146.34	**<0.001**
Fur-rubbing	−9.31	15.72	−40.13 to 21.51	0.554
Exogenous Oxytocin	−26.90	15.65	−57.57 to 3.77	0.086
N_Subject_	5			
Marginal R^2^/Conditional R^2^	0.021/0.021			

Full vs. null-model χ^2^(2) = 1.68, P = 0.431.

For the categorical predictor of oxytocin condition, the referent category is the saline condition. Significant estimates and their p-values are bolded. Full vs. null-model χ^2^ (2) = 3.02, P = 0.387.

## Discussion

Overall, we did not find that capuchin monkeys looked more or less at the eye region than the mouth region of conspecific images during the dominance categorization task, regardless of oxytocin condition. However, oxytocin condition had an impact on gaze behavior to the whole face as well as to the eye region when controlling for the stimulus image. Monkeys were significantly more likely to look at the face as a whole in the fur-rubbing condition as compared to the saline condition, and when they did look at the whole face, they did so for significantly more time in the fur-rubbing condition as compared to the exogenous oxytocin condition, suggesting that endogenous oxytocin and exogenous oxytocin impacted gaze behavior differently while monkeys were completing the task.

In addition, monkeys were significantly more likely to look at the eye region in the fur-rubbing condition than in the saline condition or the exogenous oxytocin condition, suggesting that the likelihood of looking at the eyes may be driving the similar effect of fur-rubbing on whole-face looking time. However, counterintuitively, on trials in which they did look at the eye region, monkeys looked at the eyes for significantly less time in both the fur-rubbing condition and the exogenous oxytocin condition. Finally, oxytocin condition did not have an effect on the likelihood of looking at the mouth region or, when monkeys did look at the mouth, the amount of time that they spent doing so. Our results generally support a model in which oxytocin impacts the likelihood of looking at the eye region and the face as a whole, but indicate that endogenous oxytocin (as induced by fur-rubbing behavior) and exogenous oxytocin may impact gaze behavior differently, both in likelihood of looking at these regions and in magnitude of time spent looking at these regions.

This finding that endogenous oxytocin release might impact gaze behavior differently than exogenously-administered oxytocin is an important addition to our understanding of oxytocin’s relationship to social behavior. Our previous understanding of this relationship has been largely informed by experimental manipulations involving administration of exogenous oxytocin, as very few studies have manipulated endogenous oxytocin in order to compare its effects directly with these exogenous manipulations. This is likely due to the difficulty in reliably manipulating endogenous oxytocin release *via* social behavior. However, our results, in which only fur-rubbing, but not exogenous oxytocin administration, significantly increased the likelihood of looking at the face or the eye region suggest that future research must consider if exogenous oxytocin administration affects behavior differently than when oxytocin is naturally produced and released in the body.

Why would naturally-occurring oxytocin impact gaze behavior differently than exogenously administered oxytocin? One oft-considered possibility is that intranasally administered exogenous oxytocin might not cross the blood-brain barrier due to its large molecular size (Leng and Ludwig, 2016), although recent literature suggests that it likely does or at least increases central oxytocin in some way (Chang et al., 2012; Quintana et al., 2018). Even if it does not cross the blood-brain barrier, studies that have administered exogenous oxytocin peripherally (including intranasally), indicate that exogenous oxytocin may act in other parts of the body to induce the release of endogenous oxytocin in the brain on its own (Falke, 1989; Evans et al., 2014; Lee et al., 2018). Therefore, it seems unlikely that exogenous oxytocin administration would behave differently than endogenous release due to an inability to penetrate the blood-brain barrier, especially since intranasal administration has produced behavioral effects in at least some studies (for instance, in attention to socially-relevant signals: Schulze et al., 2011; Domes et al., 2013; Prehn et al., 2013).

However, another possibility is that oxytocin’s effects are dose-dependent, and that our manipulation of endogenous oxytocin *via* fur-rubbing resulted in a lower “dose” of oxytocin than our exogenous manipulation. While our study is the first to reliably manipulate endogenous oxytocin *via* behavioral means, this dose-dependent effect is well-supported in the exogenous oxytocin literature in both humans and in other species. For instance, in male rats, only relatively low doses of injected oxytocin facilitated social recognition of juveniles while, counterintuitively, high doses resulted in impeded recognition (Popik et al., 1992). There are also dose-dependent differences in the development of parental behaviors after neonatal oxytocin injection in female prairie voles (Bales et al., 2007), as well as in anxiety behaviors after chronic oxytocin injection in mice (Peters et al., 2014). As informed by our previous work, we used a concentration of 15 IU Pro^8^ oxytocin solution, which we nebulized at a rate of 1 mL per minute in our exogenous administration condition (Benítez et al., 2018). However, given that our previous study was focused on validating the oxytocin assay and comparing the increase in urinary oxytocin after fur-rubbing as compared to a baseline, we did not analyze the contrast between the fur-rubbing condition and the exogenous oxytocin condition. Upon returning to the data from this previous study, we found that not only did both fur-rubbing and exogenous oxytocin administration significantly increase urinary oxytocin above baseline (as previously reported), but upon running this contrast, we found that exogenous oxytocin did so more than in the fur-rubbing condition (with exogenous oxytocin as the referent, β = −0.27, *SE* = 0.13, *t* = −2.06, *p* = 0.042; unpublished data, but also see Benítez et al., 2018). Therefore, it might not be that endogenously-produced oxytocin and exogenously-administered oxytocin behave differently at a mechanistic level, but rather that the manipulations result in different circulating levels of oxytocin which, in turn, impact behavior differently. This would be an important consideration to add to our understanding of oxytocin’s effects on social behavior, as behaviorally-induced endogenous oxytocin levels are likely more comparable to those resulting from natural affiliative behavior. Future research in this area could consider a dose-dependent approach to exogenous oxytocin manipulation to explore this possibility, and might include an analysis of urinary oxytocin output to compare to oxytocin levels observed after fur-rubbing.

Considering the effects of oxytocin on behavior, we did not find an overall difference in the amount of time spent attending to the eye region as compared to the mouth region in our capuchin monkeys at the session level, which differs from previous results from bonobos, who attend significantly more to the eye region than the mouth region; however, this lack of preference is similar to chimpanzees, who also do not show a preference toward one or the other (Kano et al., 2015). As the previous study of oxytocin and gaze behavior in the *Pan* species suggested that oxytocin exacerbating existing preferences (Brooks et al., 2021), it is unsurprising that oxytocin did not have an overall effect on looking time to these regions throughout the session in our study with the capuchins, as they did not exhibit a difference in looking time at all.

However, our study differed from the *Pan* study in several ways, which may make direct comparison difficult. In the previous studies, bonobo and chimpanzee subjects passively observed a mixture of videos and images over the course of 3–6 mins, whereas in our study, monkeys were actively engaged in a task with the goal of categorizing 2D still images. We intentionally chose an active task requiring still images (rather than passive viewing of videos/images) to keep our monkeys, who are accustomed to interacting with images on monitors, more engaged, however the difference in stimulus type may explain the low magnitude of looking times generally, as eyetracking studies in other primate species found that videos increased looking times in other monkey species as compared to still images (Rhesus macaques, *Macaca mulatta*, and titi monkeys, *Callicebus cupreus*: Ryan et al., 2019). Future work should explore how capuchins, particularly those from other labs less accustomed to interacting with computer monitors, and thereby potentially more engaged by passive observation, respond when viewing video footage. In addition, the capuchin monkeys in the present study had only 2 s in each trial during which we recorded their gaze behavior; while we chose to limit our gaze recording to this time period both to standardize the possible maximum looking time and to avoid distraction by the choice images, this did limit our dataset by increasing the number of trials in which monkeys looked at only one or neither of the regions of interest. Finally, because our study consisted of randomly presented stimuli, we needed to account for the stimuli presented within a session and the familiarity of the individuals within those images.

Considering how capuchins compare to other species, our monkeys did not seem to avoid eye contact, but they also did not prefer to look at the eye region over the mouth region. This is more in line with chimpanzees, which did not show a preference in the previous studies, than bonobos, who looked significantly longer at the eye region (Kano et al., 2015; Brooks et al., 2021). This is not necessarily surprising as wild capuchins live in male-dominated, mixed sex groups with a strict dominance hierarchy (Fragaszy et al., 2004b), much like chimpanzees (Parish, 1994; Watts, 2018). Perhaps in a male-dominant social environment, it’s equally important to pay attention to both the eye gaze of a male conspecific (so that one can assess their attention) and the mouth (to assess cues of potential aggression). More work could help determine if male-dominance in social organization reliably leads to this pattern of gaze allocation. In addition, rather than eyes or mouths, chimpanzees are attentive to body regions and action-target items, like toys handled by conspecifics (Kano et al., 2015); it would be interesting to explore whether capuchins would also be particularly attentive to these regions, especially given their notorious interest in manipulating objects in their environment both in the wild (Fragaszy et al., 2004a) and in captivity (Boinski et al., 1999; Brunon et al., 2014).

Of course, this lack of preference may not reflect a similarity with chimpanzees, but some other factor. For instance, it might be due to the task, which focused on dominance categorization; as we do not know which features are most relevant to dominance for capuchins (or indeed, if the face is simply considered holistically), this activity may have impacted their looking time to one or both of the regions during the 2 s that the AOIs were active on each trial. Finally, of course, our sample consists of only five individuals, an extremely small sample size that limits our statistical power and ability to generalize our conclusions. Unfortunately, the combination of requiring monkeys who are both trained to sit still for an unrestrained 60-s nebulization procedure and could be calibrated on the eyetracker (also while unrestrained) necessarily limited our sample.

While capuchins did not preferentially look at the eye or the mouth regions of conspecific faces across our three conditions, endogenous oxytocin increased the likelihood of looking at the eye region at a trial level as compared to the saline condition, in which we were able to control for the effect of individual stimuli. Intriguingly, on trials in which monkeys did look at the eyes at all, both endogenous and exogenous oxytocin significantly decreased the amount of time spent looking at the eyes. These results suggest that consideration must be given to possible differences in effects of endogenously-produced and exogenously-administered oxytocin, and that these differences should be explored to assess if they are the result of a mechanistic action difference or a dose-dependent response.

## Data availability statement

The datasets presented in this study can be found in online repositories. The names of the repository/repositories and accession number(s) can be found below: Open Science Framework: https://osf.io/sz7x2/.

## Ethics statement

This animal study was reviewed and approved by Georgia State University Institutional Animal Care and Use Committee (IACUC).

## Author contributions

MS designed the study, conducted data collection and preparation, performed statistical analyses, and wrote the manuscript. FK designed the study, provided statistical analysis guidance, and edited the manuscript. SB designed the study and edited the manuscript. All authors contributed to the article and approved the submitted version.
